# A Population-Based Surveillance Study of Shared Genotypes of Escherichia coli Isolates from Retail Meat and Suspected Cases of Urinary Tract Infections

**DOI:** 10.1128/mSphere.00179-18

**Published:** 2018-08-15

**Authors:** Reina Yamaji, Cindy R. Friedman, Julia Rubin, Joy Suh, Erika Thys, Patrick McDermott, Melody Hung-Fan, Lee W. Riley

**Affiliations:** a School of Public Health, Division of Infectious Diseases and Vaccinology, University of California, Berkeley, California, USA; b Enteric Diseases Epidemiology Branch, Centers for Disease Control and Prevention, Atlanta, Georgia, USA; c NARMS at U.S. Department of Health & Human Services/FDA, Laurel, Maryland, USA; d Public Health Laboratory Services, Contra Costa Public Health Lab, Martinez, California, USA; Antimicrobial Development Specialists, LLC

**Keywords:** *Escherichia coli*, extraintestinal diseases, molecular epidemiology, multidrug resistance, multilocus sequence typing, urinary tract infection, uropathogenic *E. coli*

## Abstract

There is increasing evidence that retail food may serve as a source of Escherichia coli that causes community-acquired urinary tract infections, but the impact of this source in a community is not known. We conducted a prospective, population-based study in one community to examine the frequency of recovery of uropathogenic E. coli genotypes from retail meat samples. We analyzed E. coli isolates from consecutively collected urine samples of patients suspected to have urinary tract infections (UTIs) at a university-affiliated health service and retail meat samples from the same geographic region. We genotyped all E. coli isolates by multilocus sequence typing (MLST) and tested them for antimicrobial susceptibility. From 2016 to 2017, we cultured 233 E. coli isolates from 230 (21%) of 1,087 urine samples and 177 E. coli isolates from 120 (28%) of 427 retail meat samples. Urine samples contained 61 sequence types (STs), and meat samples had 95 STs; 12 STs (ST10, ST38, ST69, ST80, ST88, ST101, ST117, ST131, ST569, ST906, ST1844, and ST2562) were common to both. Thirty-five (81%) of 43 meat isolates among the 12 STs were from poultry. Among 94 isolates in the 12 STs, 26 (60%) of 43 retail meat isolates and 15 (29%) of 51 human isolates were pan-susceptible (*P <* 0.005). We found that 21% of E. coli isolates from suspected cases of UTIs belonged to STs found in poultry. Poultry may serve as a possible reservoir of uropathogenic E. coli (UPEC). Additional studies are needed to demonstrate transmission pathways of these UPEC genotypes and their food sources.

**IMPORTANCE** Community-acquired urinary tract infection caused by Escherichia coli is one of the most common infectious diseases in the United States, affecting approximately seven million women and costing approximately 11.6 billion dollars annually. In addition, antibiotic resistance among E. coli bacteria causing urinary tract infection continues to increase, which greatly complicates treatment. Identifying sources of uropathogenic E. coli and implementing prevention measures are essential. However, the reservoirs of uropathogenic E. coli have not been well defined. This study demonstrated that poultry sold in retail stores may serve as one possible source of uropathogenic E. coli. This finding adds to a growing body of evidence that suggests that urinary tract infection may be a food-borne disease. More research in this area can lead to the development of preventive strategies to control this common and costly infectious disease.

## Abstract (precis)

Community-acquired urinary tract infection caused by Escherichia coli is one of the most common infectious diseases in the United States, affecting approximately seven million women and costing approximately 11.6 billion dollars annually. In addition, antibiotic resistance among E. coli bacteria causing urinary tract infection continues to increase, which greatly complicates treatment. Identifying sources of uropathogenic E. coli and implementing prevention measures are essential. However, the reservoirs of uropathogenic E. coli have not been well defined. This study demonstrated that poultry sold in retail stores may serve as one possible source of uropathogenic E. coli. This finding adds to a growing body of evidence that suggests that urinary tract infection may be a food-borne disease. More research in this area can lead to the development of preventive strategies to control this common and costly infectious disease.

## INTRODUCTION

Community-acquired urinary tract infection (CA-UTI) can greatly impact the quality of life of affected individuals and cause considerable economic burden (1–4). From the U.S. registry data, Taur and Smith estimated that seven million patients seek health care for uncomplicated UTIs in the United States each year (1). Uropathogenic Escherichia coli (UPEC) bacteria represent 80% of the pathogens that cause CA-UTI (4).

Escherichia coli strains that cause infections outside the intestinal tract such as UTIs, bloodstream infections (BSI), meningitis, and wound infections are referred to as extraintestinal pathogenic E. coli (ExPEC) (5). Intestinal pathogenic E. coli (IPEC), such as E. coli O157:H7, and other Shiga toxin-producing E. coli (STEC), are well-recognized major food-borne pathogens, but the sources of ExPEC have remained undefined. The widespread dissemination of a single lineage of uropathogenic E. coli in the United States in 1999 to 2000 led to a suggestion that CA-UTI may be a food-borne disease (6).

Subsequently, a growing number of studies have suggested that nonhuman reservoirs, especially food products, might be an important source of UPEC (7–13). Ramchandani et al. demonstrated that one E. coli isolate from beef shared 95% similarity by pulsed-field gel electrophoresis (PFGE) genotyping test to that of a human UPEC strain belonging to ST69 defined by multilocus sequence typing (MLST) (8). A study conducted in the Netherlands in 2011 identified TEM-producing ST10 UPEC from human urine samples and chicken (10). In Canada, E. coli ST117 and 131 isolates with related PFGE profiles have been identified in human infections and poultry, and ST95 was separately identified in human UTI cases and in a honeydew melon (7). Another study in 2011 also identified extended-spectrum β-lactamase (ESBL)-producing E. coli ST10 from retail meats, rectal swabs from healthy humans, and blood cultures (14). Despite these reports, the impact and magnitude of food as a source of UPEC or ExPEC are not well established.

The objective of this study was to determine the frequency of recovery of ExPEC genotypes from retail meat samples collected from the same geographic region where human cases of CA-UTIs were studied. Here, we assessed the impact of retail meat as a potential source of UPEC causing CA-UTIs in one university community by concurrently comparing the genotypes of E. coli strains isolated from urine and meat samples obtained from retail stores in Northern California counties surrounding the university community in 2016 to 2017.

## RESULTS

### E. coli isolates from human urine samples.

Between September 2016 and May 2017, we collected 1,087 nonduplicate urine samples. E. coli was isolated from 230 (21%) of these samples, yielding 233 E. coli isolates. Of these isolates, 225 were assigned to 61 unique STs; 8 could not be classified into a known ST (see Fig. 2A and see Data Set S1 in the supplemental material).

10.1128/mSphere.00179-18.1 DATA SET S1  Number of E. coli isolates with designated genotype from patients with UTIs and retail meat products in Northern California in 2016 to 2017. Among E. coli isolates from retail meat samples, seven isolates, including two from turkey, three from chicken, and two from pork could not be assigned an ST designation. Among E. coli isolates from human urine samples, eight isolates could not be assigned an ST designation. The isolates with no ST designation are not contained in the data set. Download DATA SET S1, XLSX file, 0.01 MB. Copyright © 2018 Yamaji et al. 2018 Yamaji et al. This content is distributed under the terms of the Creative Commons Attribution 4.0 International license.

### E. coli isolates from retail meat products.

**FIG 1:**
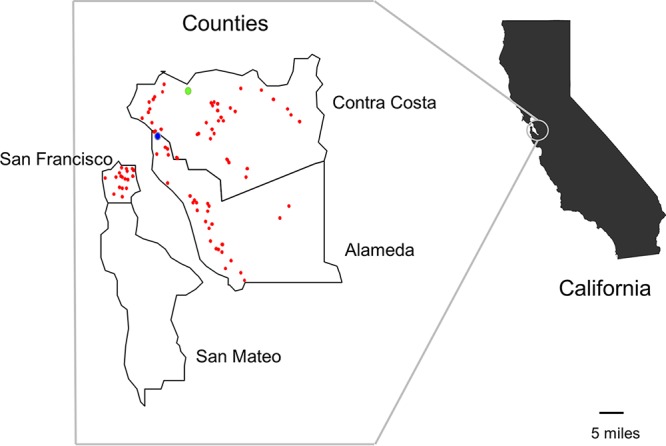
Geographic distribution of local retail markets in Northern California, where retail meat product sampling was performed in 2016 to 2017. The light-green circle shows the location of the Contra Costa County Public Health Laboratory. The blue circle represents the location of the university-affiliated health care service. Red circles represent the locations of local markets where meat samples were purchased.

Between November 2016 and September 2017, we obtained 427 retail raw meat samples; 171 chicken samples (33 breast samples, 79 leg samples, 41 wing samples, 17 thigh samples, and one sample from unknown chicken part), 87 ground turkey samples, 84 pork chop samples, and 85 ground beef samples from 68 markets in the Northern California counties of San Francisco, Alameda, and Contra Costa (Fig. 1). From 427 samples, 120 (28%) yielded E. coli; 43 (49%) from ground turkey, 45 (26%) from chicken, 18 (21%) from pork chop, and 14 (17%) from ground beef. The proportion of the samples containing E. coli was higher in poultry (chicken and turkey) than in beef samples (*P <* 0.05).

**FIG 2:**
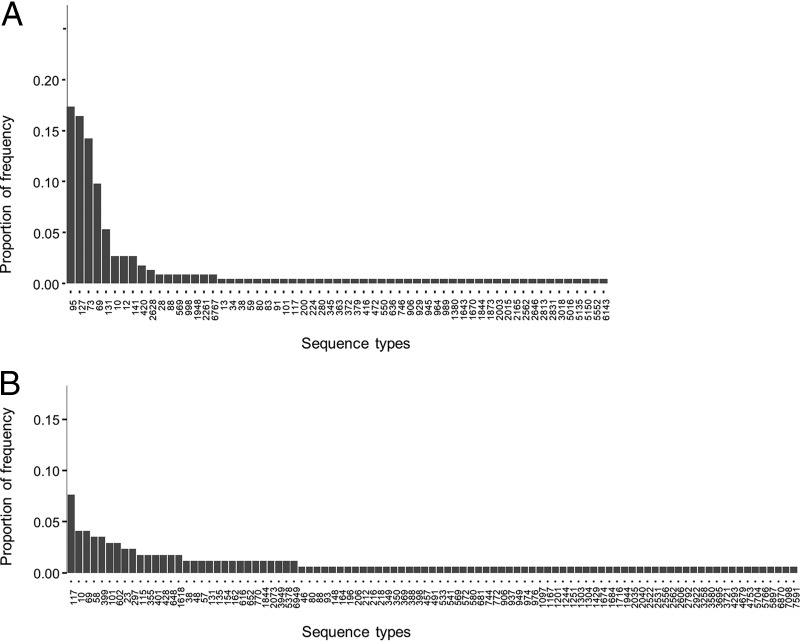
Distribution of multilocus sequence types of E. coli isolates obtained in Northern California from patients suspected to have UTI seen at the university health service (A) and from retail meat products (B).

From the 43 ground turkey samples containing E. coli, 67 E. coli isolates were recovered; 28 samples contained one genotype each, 10 samples contained two genotypes each, two samples contained three genotypes each, two other samples contained four genotypes each, and one sample contained five distinct genotypes (Data Set S2). From 45 chicken samples, 63 E. coli isolates were recovered. From 18 pork chop samples, 31 E. coli isolates were recovered. From 14 ground beef samples, 16 E. coli isolates were recovered. Thus, 177 E. coli isolates from retail meat samples were available for further analyses; 170 isolates were assigned to 95 unique STs, and seven could not be assigned any ST designation (Data Set S1, Fig. 2B, and Data Set S2).

10.1128/mSphere.00179-18.2 DATA SET S2  Number of genotypes of E. coli isolates contained in a single sample for each type of meat. Download DATA SET S2, XLSX file, 0.01 MB. Copyright © 2018 Yamaji et al. 2018 Yamaji et al. This content is distributed under the terms of the Creative Commons Attribution 4.0 International license.

### Distribution of MLST genotypes of urine and meat E. coli isolates.

The most common STs among E. coli isolates from urine samples were ST95, ST127, ST73, ST69, ST131, and ST10. These STs were found in >60% of all urine isolates collected in our study. The most common genotypes among E. coli isolates from meat were ST117, ST10, ST69, ST58, ST399, and ST101. These six genotypes were found in approximately 20% of E. coli isolates from meat isolates (Fig. 2). The values for Simpson’s diversity index were 0.91 for the urine isolate genotypes and 0.99 for the meat isolate genotypes.

**FIG 3:**
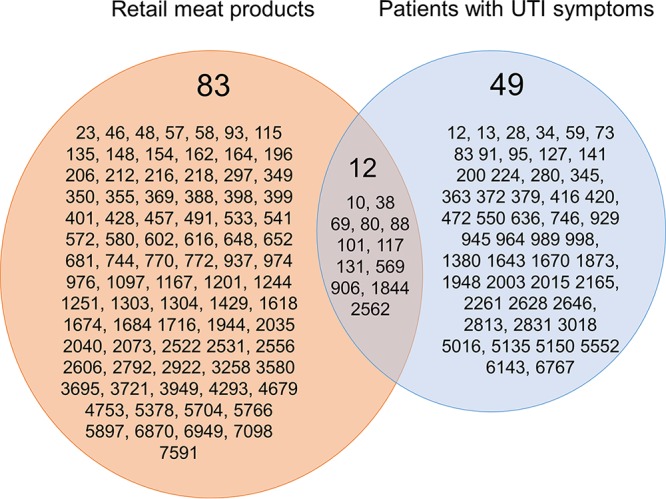
Venn diagram of the multilocus sequence genotypes of E. coli isolates obtained in Northern California from retail meat products and patients with urinary tract infections.

Twelve genotypes were shared by urine and meat E. coli isolates—ST10, ST38, ST69, ST80, ST88, ST101, ST117, ST131, ST569, ST906, ST1844, and ST2562 (Fig. 3). Of the 12 genotypes (hereafter referred to as 12 shared genotypes), ST10, ST69, and ST131 were among the most common lineages in human urine E. coli isolates. Of 83 genotypes unique to retail meat isolates, 13 (ST23, ST46, ST48, ST58, ST93, ST297, ST355, ST398, ST533, ST648, ST652, ST976, and ST1304) have been reported previously from human ExPEC isolates in the database (https://enterobase.warwick.ac.uk/species/index/ecoli).

Forty-three E. coli isolates from retail meat had the 12 shared genotypes (Table 1); 16 isolates (37%) were ST10, ST69, or ST131, and 35 (81%) were isolated from poultry (Table 1). The most common genotypes, ST117, ST10, and ST69 among E. coli from retail meat were also found in human E. coli isolates (Fig. 2 and Table 1). Overall, 51 (22%) of 233 urine isolates belonged to STs found in meat isolates.

**TABLE 1:** E. coli isolates belonging to the 12 MLST genotypes found in both human urine and retail meat samples

Sequence type	No. of *E. coli* isolates belonging to the MLST genotype					
	Retail meat samples^a^					Human urine samples
	Turkey	Chicken	Pork	Beef	All meat	
ST117	4	8	1	0	13	1
ST10	2	2	2	1	7	6
ST69	5	2	0	0	7	22
ST101	0	3	2	0	5	1
ST38	0	2	0	0	2	1
ST131	0	2	0	0	2	12
ST1844	1	1	0	0	2	1
ST80	1	0	0	0	1	1
ST88	1	0	0	0	1	2
ST569	0	1	0	0	1	2
ST906	0	0	1	0	1	1
ST2562	0	0	1	0	1	1

Total	14 (32.6)	21 (48.8)	7 (16.3)	1 (2.3)	43 (100)	51

a Numbers in parentheses represent the percentage of isolates from each type of meat.

### Phylogenetic relationship of STs of urine and meat E. coli isolates.

**FIG 4:**
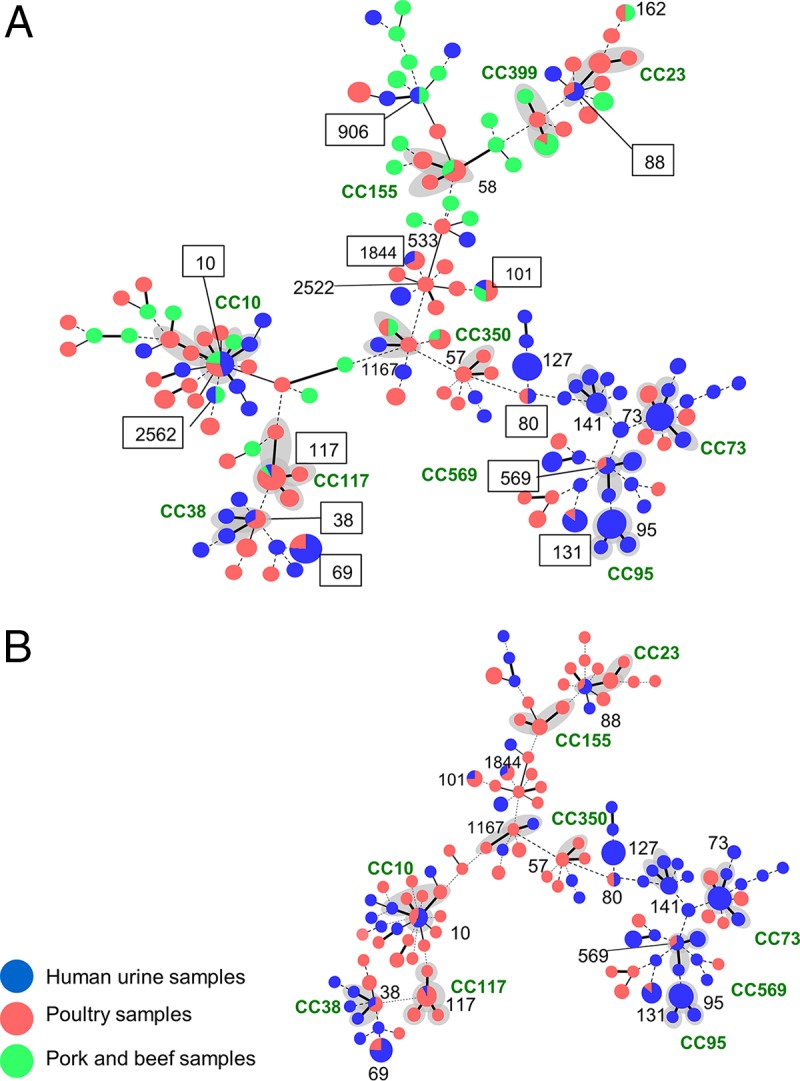
Population snapshot of E. coli isolates obtained in Northern California from patients suspected to have UTIs seen at the university health service and retail meat products. Genetic relationships among 395 E. coli isolates from human urine samples and retail meat products were visualized by the goeBURST algorithm based on the PHYLOViZ software (http://www.phyloviz.net/). Each circle represents a distinct genotype; the size of a circle is proportional to the number of isolates. Representative STs are shown as numbers without the ST prefix. The numbers in squares represent the shared genotypes (sequence type without the ST prefix) between urine and meat E. coli isolates. STs that are single-locus variants are connected with thick black lines. STs that are double-locus variants are connected with thin black lines. STs that are different at three or more loci are connected with dotted lines. Gray shading indicates that more than two STs belong to one clonal complex. (A) Isolates from three source groups (human urine samples, poultry meat samples, and pork and beef samples). (B) Isolates from two source groups (human urine samples and poultry meat samples).

We compared the E. coli multilocus sequence types for putative phylogenetic relationship of the STs based on a minimal spanning tree using globally optimized eBURST (goeBURST) (15, 16) (Fig. 4A). A distinct separation of clusters of STs by nonpoultry meat (green) and human (blue) E. coli STs is observed, whereas the STs of the isolates from poultry (red) are distributed across the entire spectrum of the goeBURST diagram. The human isolates cluster more frequently with the poultry isolates (Fig. 4A). Figure 4B shows the distribution of STs shared by poultry and human isolates. Putative founder STs included both urine and poultry isolates belonging to clonal complex 10 (CC10), CC38, CC88, CC117, and CC569, while founder STs that contained only human isolates belonged to CC73 and CC95. Single-locus variants were considered to belong to the same CC and are connected with thick black lines in the figures. Another poultry CC strain (1167) was connected to human isolates by a solid thick black line. Thus, at the CC level, one more potentially related lineage could be observed.

### Antimicrobial susceptibility of urine and meat E. coli isolates belonging to the 12 shared genotypes.

We tested isolates belonging to the 12 shared genotypes for antimicrobial susceptibility. Overall, among the 12 shared MLST genotype isolates, 27 (60%) of 43 meat E. coli isolates and 15 (29.4%) of 51 human isolates were pan-susceptible (*P <* 0.005). Resistance phenotypes found in meat isolates were found in human isolates from four genotypes (ST10, ST69, ST38, and ST131) (Table 2). For example, E. coli isolates from both meat and urine samples with ST10 were resistant to ampicillin and trimethoprim-sulfamethoxazole (TMP-SMZ). However, with ST10, resistance to gentamicin was found only among human isolates. Among the meat and urine E. coli isolates belonging to ST101 and ST569, resistance to ampicillin was found only in human isolates, and all the meat isolates were pan-susceptible. All meat and urine E. coli isolates belonging to ST1844, ST80, ST906, and ST2562 were pan-susceptible.

**TABLE 2:** Antimicrobial drug susceptibility of E. coli isolates of the 12 genotypes shared by human urine and retail meat samples^a^

Genotype (total no. of isolates typed)	Human urine samples		Retail meat samples		*P* value
	No. of pan- susceptible isolates^b^	Drug resistance^c^ (no. of isolates)	No. of pan- susceptible isolates	Drug resistance (no. of isolates)	
ST117 (14)	1	0	9	AMP (1)	
				GEN (2)	
				TMP-SMZ+GEN (1)	1.00
ST10 (13)	2	AMP (2)	5	AMP (1)	
		AMP+TMP-SMZ (1)		TMP-SMZ (1)	0.29
		AMP+CTX+TMP-SMZ+GEN (1)			
ST69 (29)	4	AMP (3)	1	AMP (6)	
		TMP-SMZ (1)			
		AMP+TMP-SMZ (11)			
		AMP+TMP-SMZ+GEN (1)			1.00
		AMP+TMP-SMZ+FOS (1)			
		AMP+TMP-SMZ+GEN+CTX+CIP (1)			
ST101 (6)	0	AMP (1)	5	0	0.17
ST38 (3)	0	AMP+CTX+TMP-SMZ+NIT (1)	0	AMP (1)	
				NIT (1)	1.00
ST131 (14)	3	AMP+CIP (2)	1	GEN (1)	
		AMP+GEN (1)			
		AMP+TMP-SMZ+GEN (1)			0.51
		AMP+TMP-SMZ+CIP (1)			
		AMP+CIP+GEN+CTX+CAZ (3)			
		AMP+CIP+GEN+CTX+CAZ+TMP-SMZ (1)			
ST1844 (3)	1	0	2	0	1.00
ST80 (2)	1	0	1	0	1.00
ST88 (3)	0	AMP (2)	0	AMP+GEN (1)	1.00
ST569 (3)	1	AMP (1)	1	0	1.00
ST906 (2)	1	0	1	0	1.00
ST2562 (2)	1	0	1	0	1.00

Total	15	36	27	16	0.002

a Antimicrobial drug abbreviations: AMP, ampicillin; CAZ, ceftazidime; CIP, ciprofloxacin; CTX, cefotaxime; GEN, gentamicin; NIT, nitrofurantoin; TMP-SMZ, trimethoprim-sulfamethoxazole.

b Pan-susceptible isolates were defined as those susceptible to all nine antimicrobial agents tested: AMP, TMP-SMZ, CIP, CTX, FOX, CAZ, NIT, FOS, and GEN.

c Resistant isolates contain those resistant to at least one antimicrobial agent of nine antimicrobial agents tested: AMP, TMP-SMZ, CIP, CTX, FOX, CAZ, NIT, FOS, and GEN. Resistance to specific antimicrobial agents is indicated.

### β-Lactamase gene types among ampicillin-resistant strains of the 12 shared genotypes.

Ampicillin-resistant ST10 and ST69 E. coli strains containing *bla*_TEM_ were found among both meat and human isolates (Table 3). Ampicillin-resistant meat and human ST38 isolates did not carry any *bla*_TEM_-type, *bla*_CTX-M_-type, *bla*_OXA_-type, *bla*_SHV_-type, or *bla*_AmpC_-type genes. Among ampicillin-resistant ST88 isolates, *bla*_TEM_-type genes were found only in a human isolate. Ampicillin-resistant ST101, ST131, and ST569 E. coli strains harboring any β-lactamase gene were found only among human isolates.

**TABLE 3:** β-Lactamase gene types identified among ampicillin-resistant E. coli isolates of the 12 shared genotypes

Sequence type	β-Lactamase gene type (no. of isolates)^a^	
	Human urine samples	Retail meat products
ST117	NA	*bla*_TEM_ type (1)
ST10	*bla*_TEM_ type (2)	*bla*_TEM_ type (1)
ST69	*bla*_TEM_ type (14)	*bla*_TEM_ type (6)
	*bla*_TEM_ type + *bla*_CTX-M_ group 1 (1)	
	Other (2)	
ST101	*bla*_TEM_ type + *bla*_CTX-M_ group 1 (1)	NA
ST38	Other (1)	Other (1)
ST131	*bla*_CTX-M_ group 1 + *bla*_OXA_ type (4)	NA
	*bla*_TEM_ type + *bla*_CTX-M_ group 9 (1)	
	*bla*_OXA_ type (1)	
	*bla*_TEM_ type (3)	
ST88	*bla*_TEM_ type (1)	Other (1)
	Other (1)	
ST569	*bla*_TEM_ type (1)	NA

a NA, not applicable. Other denotes ampicillin-resistant isolates that did not have any *bla*_TEM_-type, *bla*_CTX-M_-type, *bla*_OXA_-type, *bla*_SHV_-type, or *bla*_AmpC_-type genes. The *bla* types and genes included in the *bla* types follow: *bla*_TEM_ type, *bla*_TEM-1_ and *bla*_TEM-2_; *bla*_SHV_ type, *bla*_SHV-1_; *bla*_CTX-M_ group 1, *bla*_CTX-M-1_, *bla*_CTX-M-3_, and *bla*_CTX-M-15_; *bla*_CTX-M_ group 2, *bla*_CTX-M-2_; *bla*_CTX-M_ group 9, *bla*_CTX-M-9_ and *bla*_CTX-M-14_; *bla*_CTX-M_ group 8/25, *bla*_CTX-M-8_, *bla*_CTX-M-25_, *bla*_CTX-M-26_, and *bla*_CTX-M-39_ to *bla*_CTX-M-41_; *bla*_OXA_ type, *bla*_OXA-1_, *bla*_OXA-4_, and *bla*_OXA-30_.

## DISCUSSION

We prospectively analyzed the genotypes and antimicrobial susceptibility of E. coli isolates from human urine samples and retail meat products that were concurrently collected in the same geographic region of Northern California. We found 12 genotypes shared among human urine and meat samples. E. coli isolates from meat samples showed a greater genotypic diversity than UTI isolates. More than 80% of meat isolates that shared genotypes with human isolates were isolated from poultry sources. Of the 12 shared genotypes, ST10, ST69, and ST131 were among the most common lineages in human urine E. coli isolates. They accounted for 17% of E. coli isolates from urine samples and 10% of those from poultry samples. These STs are recognized worldwide as pandemic lineages of ExPEC (17–20).

Our findings are consistent with observations reported in other studies assessing E. coli isolates collected in a similar overlapping temporal sampling scheme from food and human sources (7, 10, 11, 14, 21–24). Studies that concurrently compared genotypes of UTI and meat E. coli isolates from Montreal, Canada, found three STs shared by human and meat sources; chicken was the most common source of these UPEC strains (7, 11). They found ST117 and ST131 in chicken, ST69 in pork only, and ST95 in a honeydew melon only. In our study, we found 12 STs shared between UTI and meat samples. The difference in the number of shared STs in this study compared to the Canadian study may be due to differences in source farms, meat processing, distribution, or study design. Nevertheless, it is striking that the three STs found in meat in the Canadian study were also found in meat in our study. In our study, eight STs were shared between human and chicken isolates, and 4 other human STs were shared by other meat types (Table 1). In addition, we found 13 other meat-associated E. coli genotypes documented in the MLST Enterobase database among our human UPEC isolates (https://enterobase.warwick.ac.uk/species/index/ecoli). Thus, we found 25 distinct UPEC STs shown either by this study or reported elsewhere to be isolated from meat sources.

Interestingly, human isolates were significantly more likely to be antibiotic resistant than meat isolates, although 20 (39%) of 51 human isolates and 29 (67%) of 43 retail meat isolates had identical drug susceptibility patterns. The overall resistance frequency of the meat isolates was lower than that observed among ExPEC isolates from retail meats in Georgia, Maryland, Oregon, and Tennessee surveyed by Xia et al. in 2006 (25). Changes in the management of antibiotics in animal husbandry over time may have contributed to the differences in resistance frequency of E. coli isolates from retail meats.

All the β-lactamase genes observed among meat isolates were identical and seen among human isolates, but not the other way around, suggesting the direction of transmission of UPEC is mainly from meat to people. A variety of food and environmental sources contain saprophytic organisms that can harbor mobile drug resistance genes (26, 27), and thus, it is conceivable that the human intestine is routinely colonized with bacteria that carry such genes. Certainly, there has been considerable interest in the gut microbiota as a potential source of antimicrobial resistance (“the gut resistome”) (28–31). For example, Sommer et al. found that nearly half of the resistance genes in cultured aerobic gut isolates were identical to resistance genes harbored by major pathogens (31). If so, it may be speculated that drug-susceptible meat strains may acquire drug resistance genes in the human intestine.

Phylogenetic analyses by goeBURST showed clustering of human isolates with poultry isolates (Fig. 4). Many of the STs assigned as founder STs contained both human and poultry isolates, which precluded determination of the direction of transmission. Single-locus variants linked to the founder STs included strains from human and poultry sources also, and thus, they did not provide information needed to suggest transmission direction. A larger sample size or comparison by a higher-resolution genotyping method such as whole-genome sequencing may be needed to demonstrate with more confidence if poultry is a source of human UPEC. Nevertheless, given the large number of STs and the well-validated application of MLST to depict phylogenetic relationships of E. coli (32), the observation in this population-based study of an overlap of STs of E. coli isolates from poultry and human urine samples suggests that poultry is a source of UPEC. Of a total of 121 different STs identified from both urine and poultry samples in this study, 10 (8%) overlapped.

ST95 (a pandemic ExPEC lineage) was the most common ST among UPEC isolates in this study, accounting for 39 (17%) of all the suspected cases of UTIs. We did not find any ST95 isolates in retail meat. Nevertheless, Vincent et al. reported the isolation of an ST95 strain from a honeydew melon in Canada (7), and studies in Europe documented ST95 strains from poultry (24, 33). ST95, sometimes described as avian-pathogenic E. coli (APEC), has been isolated from both domestic and wild birds (34, 35). We also did not find ST73 and ST127 (other pandemic lineages) among the meat E. coli isolates. This finding suggests that UPEC ST73, ST95, and ST127 may have other sources in the United States. Other potential sources include vegetables, fruit, seafood, or environmental sources we did not examine.

An important limitation of our study was that we did not obtain clinical, travel or food histories to link potential sources of the isolates recovered from the patients. However, we note that this study was not designed to show direct connection of the meat isolates as the causative agent of human UTIs. It was mainly designed to determine how much of the meat E. coli isolates belonged to recognized ExPEC lineages isolated from CA-UTI cases in the same geographic region. The UTI isolate collection overlapped 6 months of a 10-month meat collection period. Unlike gastrointestinal illnesses in which the timing of exposure (contaminated food ingestion) can be estimated from the incubation period, such exposure history cannot be easily documented with CA-UTI. The duration of human intestinal colonization by an E. coli genotype can vary greatly (36), and hence, the time of ingestion of a suspected food item cannot be approximated.

We also note that the demonstration of identical E. coli STs in two sources does not necessarily show disease causality. Here, disease causality is assumed for some of the STs because of their membership in recognized ExPEC STs, especially those that are pandemic. As previously mentioned, other studies have suggested food as a potential source of ExPEC. What this study attempted to do further was to assess the magnitude of food as a potential reservoir of ExPEC. This was a population-based study where E. coli bacteria from food and UTI samples were prospectively analyzed contemporaneously in the same geographic setting. We took advantage of an extensive retail meat survey being conducted by the U.S. Food and Drug Administration (FDA) in the very region where the UTI study was simultaneously conducted. To date, no other studies have identified so many ExPEC lineages in meat samples.

In conclusion, this investigation identified 12 MLST E. coli genotypes shared between urine and retail meat E. coli isolates. We found that poultry may be a common reservoir of UPEC and a potential source of its transmission. Additional research is needed to examine the importance and magnitude of food products as a source of human CA-UTIs. With ever-increasing prevalence of CA-UTIs caused by multiple-drug-resistant (MDR) UPEC strains, a better understanding of sources of UPEC could help devise novel public health control interventions to combat this common infectious disease problem.

## MATERIALS AND METHODS

### Study design.

We prospectively cultured E. coli from urine samples from patients suspected to have UTIs at a Northern California university health center and from meat products collected from retail stores in three Northern California counties (Fig. 1).

### Isolation of E. coli from urine samples.

Between 19 September 2016 and 4 May 2017, we tested urine samples that were collected consecutively from patients suspected to have UTIs seen at the university health service (Fig. 1). All urine samples were first tested at the health service by dipstick, and those specimens found to test positive for leukocytes, nitrates, protein, blood, or glucose were collected for our study and subjected to further microscopic examination. We defined a case of UTI as a symptomatic patient with clean-catch urine specimen that contained more than 10^2^ CFU of E. coli per ml. We cultured a 10-µl aliquot of urine on a MacConkey agar plate to isolate Gram-negative bacteria and presumptively identified lactose-positive and indole-positive colonies as E. coli for further analysis.

### Isolation of E. coli from retail meat products.

Between 29 November 2016 and 29 September 2017, we obtained retail raw meat samples: skin-on/bone-in chicken; chicken breast, leg, wing, and thigh; ground turkey; pork chops; and ground beef. Retail meat products were collected as part of the National Antimicrobial Resistance Monitoring System (NARMS), a national public health food surveillance system involving collaboration of state and local public health departments, Centers for Disease Control and Prevention (CDC), the U.S. Food and Drug Administration (FDA), U.S. Department of Agriculture (USDA), and Food Safety Inspection Service (FSIS) (37). Retail meat products (chicken, ground turkey, ground beef, and pork chops) were purchased at local grocery stores in California. The Contra Costa County Public Health Laboratory selected sampling zip codes that are at least within a 50-mi radius of the laboratory and purchased the retail meat samples from local grocery stores within these zip codes by random selection from a list obtained from the Northern California municipality. The university health service is located within this radius. Twice a month, the laboratory purchased a total of 40 meat samples, including 20 samples each of chicken, 10 samples of ground turkey, 5 samples of ground beef, and 5 samples of pork chops. When bone-in/skin-on chicken breasts were not available for sampling, chicken wings were used as the preferred substitution, followed by legs and thighs. The laboratory recorded information, including the store name, store location, brand name, sell-by date, purchase date, and laboratory processing date for purchased meat on monthly log records.

Retail meat products were kept on ice during transport from the local grocery stores to Contra Costa County Public Health Laboratory. Samples were refrigerated and processed within 24 h of purchase. Each retail meat sample was transported aseptically into a sterile sample bag containing 225-ml buffered peptone water. Several chicken pieces were used to achieve a 25-g sample when substituting chicken breast with another chicken part; wing, leg, or thigh. One pork chop from each retail package was aseptically placed into a sterile bag containing 225-ml buffered peptone water. Samples (25 g) of ground beef and ground turkey were aseptically transferred from each package into a sterile bag containing 225-ml buffered peptone water (38). The sealed bags were shaken by a mechanical shaker at 200 rpm for 15 min. The samples were tested for *Salmonella* and *Campylobacter* at the Contra Costa County Public Health Laboratory. After the samples were processed, we transported the retail meat samples in the sealed bags to the project laboratory at the University of California, Berkeley, for E. coli isolation.

A 15-ml aliquot of the peptone water from each bag containing meat samples was preincubated in double strength MacConkey broth in a 1:1 dilution at 35°C for 24 h. A separate 10-µl aliquot of preincubated peptone water from each bag containing meat was cultured on a MacConkey agar plate to isolate Gram-negative bacteria. Lactose-positive and indole-positive colonies were presumed to be E. coli and selected for further analysis.

### Strain typing.

Five colonies recovered on plate from each sample were randomly picked and subtyped by the ERIC2-PCR assay, as described previously (39, 40). Single colonies from tryptic soy agar plates were selected and inoculated into 2 ml tryptic soy broth and incubated in a shaking incubator for 15 h at 37°C. The 1-ml aliquots of grown cultures were centrifuged, and the pellets were resuspended in a test tube with 350 µl of distilled water, boiled for 10 min in a water bath, and then cooled on ice for 2 min. The samples were centrifuged for 2 min at 13,000 rpm, and the supernatants were stored at −20°C before they were subjected to PCR tests. The five colonies that had identical enterobacterial repetitive intergenic consensus (ERIC) electrophoretic banding patterns by visual inspection were considered to belong to the same clonal group, and one of these colonies was selected for further analysis by multilocus sequence typing (MLST). If samples contained more than one distinct electrophoretic banding pattern by ERIC2-PCR typing, all strains were included in the MLST analysis. All E. coli isolates were genotyped by MLST based on the seven-gene scheme described at website https://pubmlst.org/ (41). The allelic number and the corresponding genotype number were designated by the curator of the MLST website.

### Analysis of MLST data.

To assess possible evolutionary relationships of the sequence type (ST) lineages, we compared the allelic profiles of each ST by the globally optimized eBURST (goeBURST) algorithm maintained in PHYLOViZ 2.0 (http://www.phyloviz.net/) (15, 16). We used the stringent definition of eBURST group as all members that have identical alleles at six or seven of the seven MLST loci with at least one other member of the group. An ST with the highest number of single-locus variants (SLVs) was predicted as a putative founder ST. A cluster of linked STs was considered to represent a clonal complex (CC), shown in gray in the figures.

### Antimicrobial susceptibility testing.

Isolates from retail meat and human urine samples that had the same genotypes were assessed for susceptibility to ampicillin (AMP), trimethoprim-sulfamethoxazole (TMP-SMZ), ciprofloxacin (CIP), cephalosporins (cefotaxime [CTX], cefoxitin [FOX], and ceftazidime [CAZ]), nitrofurantoin (NIT), fosfomycin (FOS), and gentamicin (GEN) by the standard disc diffusion assay. Susceptibility cutoffs were determined according to the standard interpretive criteria of the Clinical and Laboratory Standards Institute (42). E. coli 25922 from the American Type Culture Collection was used as a reference strain. Isolates with intermediate susceptibility were classified as resistant.

### β-Lactamase gene identification.

Ampicillin-resistant E. coli isolates were examined for β-lactamase gene families by multiplex PCRs as described previously (43, 44). These β-lactamase gene families included the following: *bla*_TEM_ type (*bla*_TEM-1_ and *bla*_TEM-2_), *bla*_SHV_ type (*bla*_SHV-1_), *bla*_CTX-M_ group 1 (*bla*_CTX-M-1_, *bla*_CTX-M-3_, and *bla*_CTX-M-15_), *bla*_CTX-M_ group 2 (*bla*_CTX-M-2_), *bla*_CTX-M_ group 9 (*bla*_CTX-M-9_ and *bla*_CTX-M-14_), *bla*_CTX-M_ group 8/25 (*bla*_CTX-M-8_, *bla*_CTX-M-25_, *bla*_CTX-M-26_, and *bla*_CTX-M-39_ to *bla*_CTX-M-41_), *bla*_OXA_ type (*bla*_OXA-1_, *bla*_OXA-4_, and *bla*_OXA-30_), and *bla*_AmpC_ types (*bla*_MOX-1_, *bla*_MOX-2_, *bla*_CMY-1_, *bla*_CMY-8_ to *bla*_CMY-11_, *bla*_LAT-1_ to *bla*_LAT-4_, *bla*_CMY-2_ to *bla*_CMY-7_, *bla*_BIL-1_, *bla*_DHA-1_, *bla*_DHA-2_, *bla*_ACC_, *bla*_MIR-1T_, *bla*_ACT-1_, and *bla*_FOX-1_ to *bla*_FOX-5b_). To detect the plasmid-mediated AmpC β-lactamase genes, we performed six types of multiplex PCR as described previously (44).

### Data analysis.

Simpson’s diversity index was used to compare the diversity of STs between meat and urine E. coli isolates (45). The difference in prevalence of the genotypes and drug resistance of E. coli isolates between patients who had urine samples collected for UTI symptoms and retail meat samples was assessed by two-sided Fisher’s exact test. Statistical significance was defined as a *P* value of <0.05. All analyses were performed by R-Studio version 3.4.3.
